# Cryo-SIMPLY: A Reliable STT-MRAM-Based Smart Material Implication Architecture for In-Memory Computing

**DOI:** 10.3390/nano15010009

**Published:** 2024-12-25

**Authors:** Tatiana Moposita, Esteban Garzón, Adam Teman, Marco Lanuzza

**Affiliations:** 1 Department of Computer Engineering, Modeling, Electronics, and Systems Engineering, University of Calabria, 87036 Rende, Italym.lanuzza@dimes.unical.it (M.L.); 2 Emerging NanoScaled Integrated Circuits & Systems Labs, Faculty of Engineering, Bar-Ilan University, Ramat Gan 5290002, Israel; adam.teman@biu.ac.il

**Keywords:** STT-MRAM, SMTJ, DMTJ, magnetic tunnel junction, in-memory computing, material implication, IMPLY, SIMPLY, cryogenic, 77 K, logic-in-memory

## Abstract

This paper presents Cryo-SIMPLY, a reliable smart material implication (SIMPLY) operating at cryogenic conditions (77 K). The assessment considers SIMPLY schemes based on spin-transfer torque magnetic random access memory (STT-MRAM) technology with single-barrier magnetic tunnel junction (SMTJ) and double-barrier magnetic tunnel junction (DMTJ). Our study relies on a temperature-aware macrospin-based Verilog-A compact model for MTJ devices and a 65 nm commercial process design kit (PDK) calibrated down to 77 K under silicon measurements. The DMTJ-based SIMPLY demonstrates a significant improvement in read margin at 77 K, overcoming the conventional SIMPLY scheme at room temperature (300 K) by approximately 2.3 X. When implementing logic operations with the SIMPLY scheme operating at 77 K, the DMTJ-based scheme assures energy savings of about 69%, as compared to its SMTJ-based counterpart operating at 77 K. Overall, our results prove that the SIMPLY scheme at cryogenic conditions is a promising solution for reliable and energy-efficient logic-in-memory (LIM) architectures.

## 1. Introduction

In-memory computing (IMC) is a promising paradigm that addresses the von Neumann bottleneck of conventional computing systems [1,2,3,4]. By enabling computations directly within memory arrays, IMC avoids the need for energy/time-consuming data transfer between memory and processing units [5,6]. While traditionally implemented with conventional CMOS-based technologies [7,8], IMC architectures have also been extensively explored using non-volatile memory (NVM) technologies, such as resistive RAM (RRAM) [9,10], magnetic RAM (MRAM) [11], and ferroelectric field-effect transistors (FeFETs) [12,13,14].

Within the realm of IMC, NVM-based logic-in-memory (LIM) has emerged as a reliable, low-power, and high-performance solution [15]. Among the various LIM schemes proposed in the literature [16,17,18], material implication (IMPLY) logic has been demonstrated to be a compelling choice due to the low additional complexity required to perform logical operations directly within memory arrays. In the IMPLY approach, memory devices are used simultaneously as computing and storage elements. When performing a logic operation, inputs are pre-encoded as resistance states of memory devices, and specific voltage pulses determine if the resistance of the output memory cell changes based on the input combination. However, IMPLY-based designs suffer from reliability issues, including the degradation of logic states and severe sensitivity to driving voltage variations [19]. To address these concerns, the smart IMPLY (SIMPLY) architecture was introduced, also demonstrating enhanced energy efficiency in executing logic operations [19,20,21,22]. Although proposed for RRAM technology [19,21,23,24], IMPLY and SIMPLY logic schemes have recently been evaluated for implementation with spin-transfer torque MRAMs (STT-MRAMs) [15].

Compared to RRAM, STT-MRAM offers several benefits for the SIMPLY logic scheme, including higher energy efficiency, better write endurance, and lower bit error rates [15]. However, the STT-MRAM-based SIMPLY scheme, typically based on single-barrier magnetic tunnel junction (SMTJ), suffers from long write access times, mainly due to the relatively high switching currents [25]. Double-barrier MTJ (DMTJ) offers a potential solution for faster and more energy-efficient switching [26]. However, DMTJ presents a lower resistance ratio, which results in a reduced read margin [26], especially critical for reliable SIMPLY operation.

In this work, we propose Cryo-SIMPLY, a reliable STT-MRAM-based LIM scheme leveraging cryogenic electronics [27] and an in-memory computing paradigm [28]. More specifically, we used cryogenic operating temperatures (liquid nitrogen boiling point, 77 K) to address the limitations of DMTJ-based SIMPLY designs. Prior research [29,30] has shown that STT-MRAMs at cryogenic temperatures are a good choice for reliable and energy-efficient computing. We leveraged this concept by applying a cryogenic operation to STT-MRAM-based SIMPLY logic. Cryo-SIMPLY aims to address the memory and power limitations of conventional von Neumann computing architectures while offering a compelling solution for future computing systems.

SIMPLY designs were evaluated using our macrospin-based Verilog-A temperature-dependent compact models reported in [26] of 30 nm perpendicular STT-MTJs and a commercial cryogenic-aware 65 nm CMOS process. Through comprehensive circuit and application level simulations, we characterized and benchmarked the performance of both SMTJ and DMTJ SIMPLY designs.

The key contributions of this work are as follows:

- To our knowledge, this is the first study presenting a circuit-to-application level characterization and comparative analysis between SMTJ- and DMTJ-based SIMPLY schemes operating at 77 K.
- The DMTJ-based SIMPLY demonstrates a significant improvement in read margin at 77 K, overcoming the conventional SIMPLY scheme at room temperature (300 K) by approximately 2.3 X.
- When implementing logic operations with the SIMPLY scheme operating at 77 K, the DMTJ-based scheme assures energy savings of about 69%, as compared to its SMTJ-based counterpart operating at 77 K.
- The energy efficiency observed at cryogenic temperatures makes the DMTJ-based SIMPLY scheme a viable option for low-power LIM.

The rest of this paper is organized as follows: Section 2 provides background on hybrid CMOS/MTJ circuits operating at cryogenic temperatures. Section 3 details the SIMPLY architecture. Section 4 presents the simulation results obtained at cryogenic temperatures. Finally, Section 5 concludes the paper.

## 2. CMOS and MTJ Technologies Operating at Cryogenic Temperatures

### 2.1. CMOS Devices Under Cryogenic Operation

Standard CMOS technology can operate across a wide temperature range, but commercially available process design kit (PDK) models lose accuracy at cryogenic temperatures (below 200 K) where key transistor properties are significantly impacted [31,32]. As the operating temperature goes down to the cryogenic levels, transistors exhibit an increase in threshold voltage, carrier mobility, and saturation velocity, as well as a higher sub-threshold slope [32,33].

Early studies often relied on cryogenic-aware transistor models based on existing research or simulations [34,35,36]. In contrast, this work uses a 65 nm CMOS BSIM4.7-based transistor model, calibrated for cryogenic operation down to 77 K. The calibration was carried out via silicon measurements from liquid nitrogen-cooled test chips. Our calibrated model exhibits very high accuracy at cryogenic temperatures.

**Figure 1:**
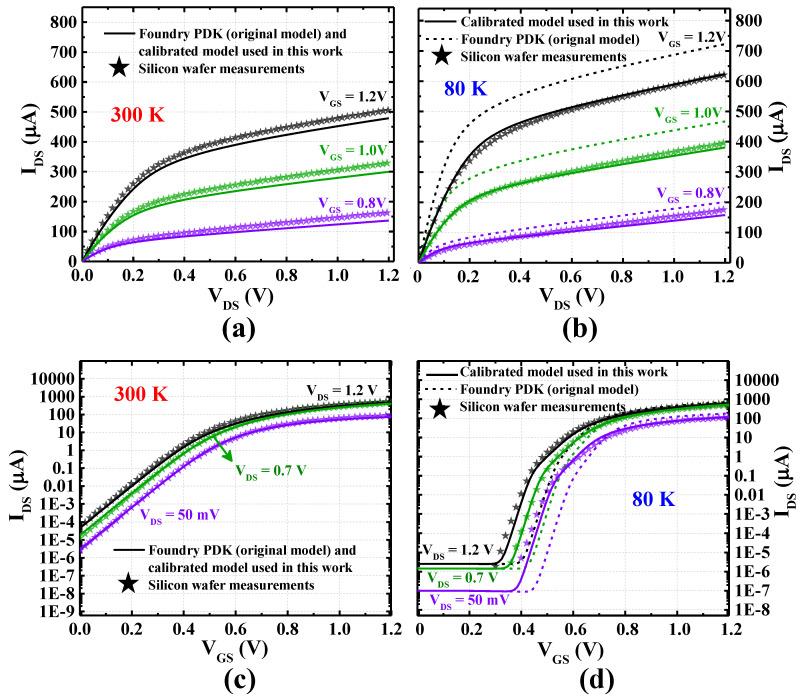
Current–voltage characteristics of CMOS transistors at 300 K and 80 K: (**a**,**b**) drain–source current (*I*_DS_) as a function of drain–source voltage (*V*_DS_) for various gate–source voltage (*V*_GS_); (**c**,**d**) *I*_DS_ as a function of *V*_GS_ for various *V*_DS_. Data refer to an nMOS transistor with a channel length of 60 nm and a width of 1 um, with the body–source voltage (*V*_BS_) set to 0 V (reproduced from our previous work [30]).

Figure 1 compares the nMOS transistor current–voltage characteristics at both room (300 K) and cryogenic (77 K) temperatures for three scenarios: the original foundry PDK model, our calibrated model, and actual silicon measurements. While both models closely match the experimental data at room temperature, the calibrated model demonstrates a substantial reduction in average error at 80 K. The non-calibrated commercial PDK model suffers from errors of about 18% and 70% for drain–source current (*I*_DS_) versus drain–source voltage (*V*_DS_) and for *I*_DS_ versus gate–source voltage (*V*_GS_), respectively. In comparison, our calibration reduces the average error in current–voltage characteristics to less than 2% for *I*_DS_ versus *V*_DS_ and less than 10% for *I*_DS_ versus *V*_GS_.

### 2.2. Magnetic Tunnel Junctions (MTJs) Operating at Cryogenic Temperatures

Several papers have experimentally demonstrated the cryogenic operating temperature of MTJ nanopillars, mainly based on different stack layers. Lang et al. evaluated the low-temperature performance of CoFeB/MgO-based perpendicular MTJs by characterizing their switching voltage, write error rate, and endurance at temperatures as low as 9 K [37]. Veiga et al. investigated the optimization of perpendicular MTJs for efficient STT switching at cryogenic temperatures [38]. Their study compared four storage layer structures: FeCoB/W, Mg/FeCoB/W, FeCoB/Ru/W, and FeCoB/W/Py/W (Py is permalloy Ni_80_Fe_20_). The inclusion of permalloy in the storage layer was shown to effectively reduce interface anisotropy, thereby lowering critical switching currents. Lyu et al. explored the development and performance of perpendicular MTJs utilizing molybdenum (Mo) as a key material [39]. The study focuses on achieving sub-nanosecond switching speed while operating at cryogenic temperatures. Cao et al. evaluated the performance of nanoscale perpendicular MTJs when operating at low temperatures [40]. Results suggest that the MTJs that present a free layer with double MgO interfaces are promising candidates for applications in cryogenic environments. Rehm et al. analyzed the spin-transfer switching characteristics of perpendicular MTJ nanopillars (CoFeB/W/CoFeB) with diameters ranging from 40 to 60 nm, when operating at temperatures down to 4 K and switching on nanosecond timescales [41].

In this work, we focus on STT-SMTJ and STT-DMTJ stacks based on CoFeB/MgO layers. These nanopillar structures are comprehensively evaluated as part of a LIM scheme operating at 77 K, as detailed in Section 3. The reliability benefits of nanopillar structures under cryogenic conditions are further discussed in Section 4.

### 2.3. Related Work

Cryogenic MTJ-based circuit schemes have gained momentum as a promising solution for enabling reliable and energy-efficient operations for future cryogenic memory systems. Hou et al. introduced a 77 K cryogenic 1 Kb STT-MRAM design using MTJ nanopillars for energy-efficiency computing [42]. In [29,43], a cryogenic STT-MRAM design operating at 77 K that leverages a DMT device with two reference layers was evaluated. This architecture provides improved energy for read and write operations, as compared to the conventional SRAM scheme. In [28], an STT-MRAM operating at 77 K showed performance and energy benefits enabled by a write trimming system. Zhang et al. designed an MRAM based on spin–orbit torque (SOT) operating below 10 K [44]. The SOT-MRAM scheme showed improvements in tunnel magnetoresistance and coercive field, leading to improved reliability for cryogenic computing applications. Nguyen et al. proposed a cryogenic memory array consisting of a non-volatile three-terminal magnetic tunnel junction element driven by the spin Hall effect, achieving reliable switching between resistance states with low error rates and energy-efficient operations [45]. Overall, MTJ-based computing architectures operating at cryogenic temperatures offer benefits, such as better reliability through higher TMR, low leakage currents, and improved data retention time.

### 2.4. Spin-Transfer Torque Magnetic Tunnel Junction (STT-MTJ) Devices and Temperature-Dependent Compact Model

**Figure 2:**
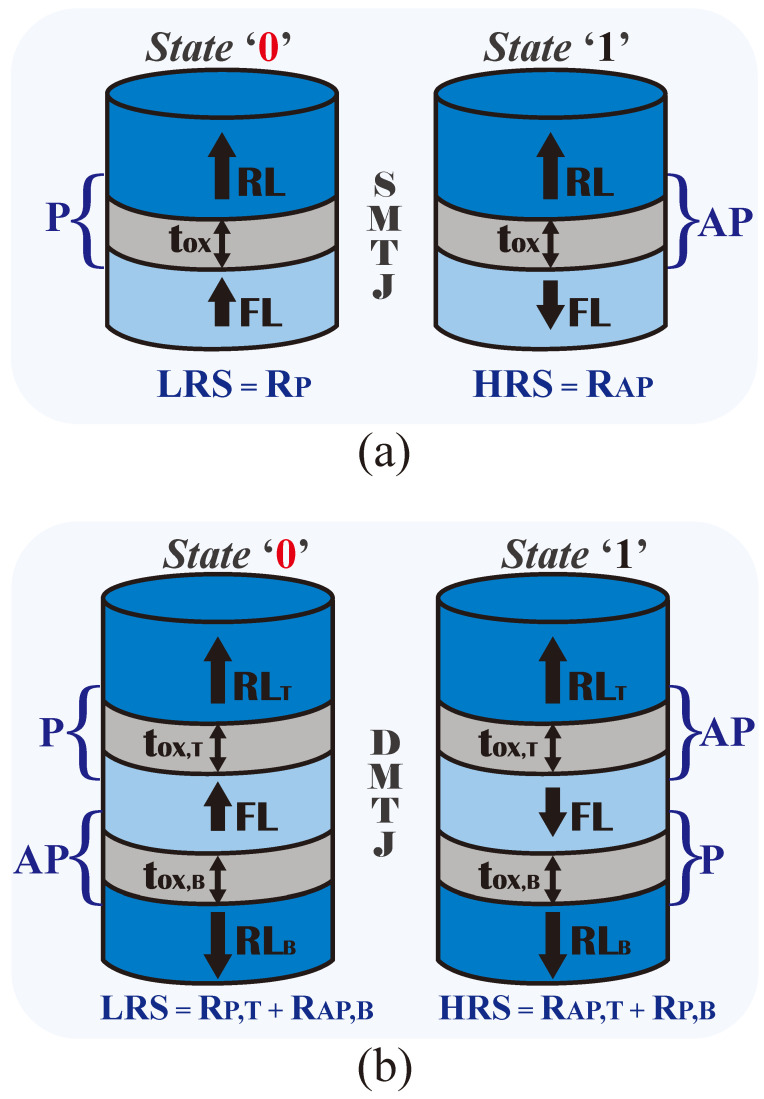
(**a**) Single-barrier magnetic tunnel junction (SMTJ) structure; (**b**) double-barrier magnetic tunnel junction (DMTJ) structure. MTJs feature low and high resistance states (LRS and HRS), corresponding to bit ‘0’ and bit ‘1’, respectively.

Figure 2 shows the sketches of the perpendicular (a) STT-SMTJ and (b) STT-DMTJ devices considered in this study. Both devices share a core structure built from ferromagnetic layers separated by thin MgO oxide barriers. For the SMTJ, the oxide barrier of thickness ($t_{\mathrm{OX}}$) is sandwiched between two CoFeB ferromagnetic layers: the reference layer (RL) and the free layer (FL). The DMTJ exhibits a more complex structure with two oxide barriers ($t_{\mathrm{OX},T}$ and $t_{\mathrm{OX},B}$) separating three ferromagnetic layers: a top reference layer (${\mathrm{RL}}_{T}$), a free layer (FL), and a bottom reference layer (${\mathrm{RL}}_{B}$). The RLs have a fixed magnetization orientation, while the FL has a variable magnetization orientation. Therefore, the two MTJ structures allow for two stable resistive states, depending on the relative magnetization orientation between RL in the case of the SMTJ (or ${\mathrm{RL}}_{T}$ and ${\mathrm{RL}}_{B}$ for the DMTJ) and FL.

Figure 2 also shows the different resistance states of the SMTJ and DMTJ devices. The SMTJ devices exhibit a low resistance state (LRS) when in the parallel (P) state and a high resistance state (HRS) when in the antiparallel (AP) state. The DMTJ resistance can be modeled as two series-connected resistances, one for each oxide barrier. This translates to different LRS and HRS values compared to the SMTJ device. For this work, the LRS and HRS correspond to bit ‘0’ and bit ‘1’, respectively.

To write information into the devices, the spin-transfer torque (STT) mechanism [46] was exploited, which uses spin-polarized current to change the magnetization state. For the SMTJ, the switching between P and AP states occurs when a current exceeding the critical switching current ($Ic_{P}$ or $Ic_{AP}$, with $Ic_{P}$ larger than $Ic_{AP}$) is injected in the proper direction. The two reference layers, with antiparallel magnetizations of the DMTJ, lead to more symmetrical switching behavior and lower switching current compared to the SMTJ [30].

We used the analytical macrospin-based Verilog-A compact models to evaluate the behavior and electrical characteristics of SMTJ and DMTJ devices. These models incorporate the cryogenic temperature dependence of key physical parameters using established equations [47,48,49].

A detailed explanation of the adopted modeling can be found in [43]. Our cryogenic-aware MTJ modeling has been validated in previous works [30].

Table 1 summarizes the main physical and electrical characteristics of the MTJ devices, where the temperature-dependent parameters are explicitly provided for both room and cryogenic temperatures. Lower temperatures increase Ic and HRS, thereby improving reliability in terms of read operation but sacrificing speed and energy during write memory access [30]. All the temperature trends are in agreement with the experimental studies previously reported in the literature [37,40,41,49]. For more detailed information regarding the temperature-dependent equations and trends in the electrical characteristics of the MTJ models, the reader is referred to [29,43]. Note that the magnetic parameters reported in [29,43] and used in our analysis mainly refer to a CoFeB FL.

## 3. Smart Material Implication (SIMPLY) Logic Scheme

The SIMPLY logic scheme stands out as a promising STT-MRAM-based LIM approach [15]. Notably, SIMPLY and sFALSE (a logic operation always resulting in 0) form a universal logic set, enabling the implementation of any logic gate using these two operations alone [21].

**Figure 3:**
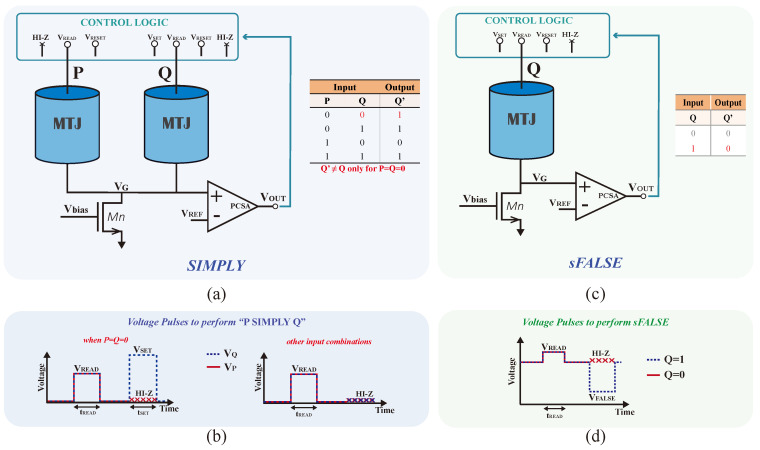
(**a**) STT-MRAM-based SIMPLY cell with control logic, tail transistor, and precharge sense amplifier (PCSA) as comparator; (**b**) the timing diagram of ‘P SIMPLY **Q**’ for **P** = **Q** = ‘0’ and in all other cases; (**c**) sFALSE scheme; (**d**) the timing diagram of ‘sFALSE **Q**’ operation when **Q** = ‘1’ (blue line) and **Q** = ‘0’ (red line).

Figure 3a shows the STT-MRAM-based SIMPLY scheme, which consists of 2 MTJs, **P** and **Q**, control logic, an nMOS tail transistor, Mn (a SIMPLY array [15] requires a single Mn transistor per row), and a precharge sense amplifier (PCSA). Inputs are pre-stored as resistance states of MTJs, whereas the operation result eventually overwrites the MTJ **Q**. SIMPLY operation is orchestrated by the control logic block, which includes analog tri-state buffers (TSBs) responsible for applying the necessary voltages to the MTJs. The read voltage (*V*_READ_), reset voltage ($V_{\mathrm{RESET}}$), set voltage ($V_{\mathrm{SET}}$), bias voltage ($V_{\mathrm{bias}}$), and reference voltage (*V*_REF_) are the voltage values set during SIMPLY and sFALSE operations. Note that $V_{\mathrm{bias}}$ voltage is used to implement a load resistor.

SIMPLY operation is based on the observation that according to the truth table for the IMPLY logic operation (shown in Figure 3a), the only input configuration that actually leads to **Q**’≠ **Q** and therefore requires MTJ **Q** to switch from ‘0’ to ‘1’ is the one for which **P** = **Q** = ‘0’, while in all the other cases, it must be **Q**’ = **Q** which makes energy-hungry MTJ **Q** switching unnecessary. So, being able to distinguish this input configuration from the others would be profitable. To accomplish this, the SIMPLY operation comprises a preliminary read, followed by a set phase (see Figure 3b). During the preliminary read operation, a $V_{\mathrm{read}}$ pulse with width $t_{\mathrm{read}}$ is applied to MTJs **P** and **Q**. This produces a *V*_G_ voltage that is compared to *V*_REF_ by a PCSA. The output ($V_{\mathrm{out}}$) of the PCSA discriminates the case **P** = **Q** = ‘0’ from all the others. In the subsequent phase, a SET pulse, with amplitude $V_{\mathrm{SET}}$ and width $t_{\mathrm{SET}}$, is applied on **Q** only when **P** = **Q** = ‘0’ to switch **Q** from ‘0’ to ‘1’. During the SET phase, **P** is connected to a high impedance (HI-Z), regardless of the result in the preliminary reading [21,22].

As shown in Figure 3c, the sFALSE operation is performed on a single MTJ device and stores the output within the same MTJ. The sFALSE execution always results in a logic ‘0’ being stored in the MTJ. Figure 3d shows the sFALSE operation applied to **Q**, i.e., ‘sFALSE **Q**’. Similar to SIMPLY operation, a *V*_READ_ pulse of width $t_{\mathrm{READ}}$ is applied to **Q** beforehand. This is followed by a RESET operation that applies a negative voltage ($V_{\mathrm{FALSE}}$) only if the state of **Q**, sensed by the PCSA, is ‘1’ [20].

**Figure 4:**
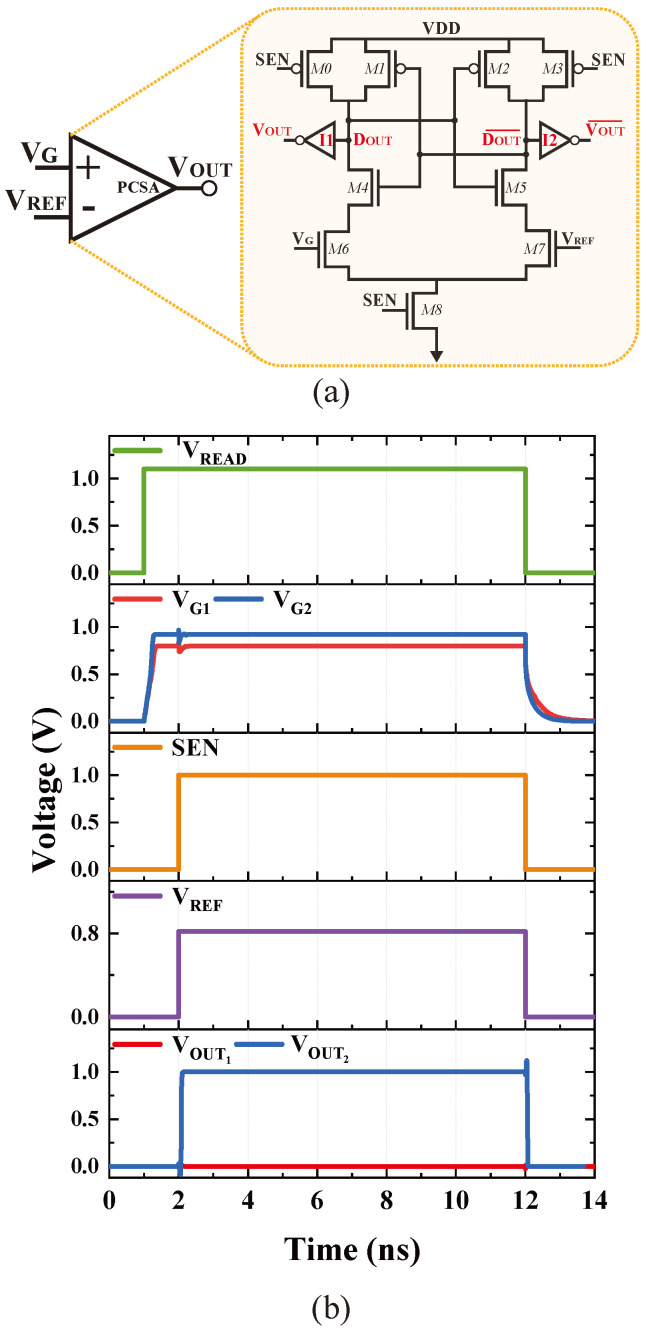
PCSA-based comparator: (**a**) schematic and (**b**) timing diagram. In (**b**), two read operations are shown: $V_{G1}$ (read **P** = **Q** = ‘0’) and $V_{G2}$ (read **P** ≠ **Q**).

Figure 4a shows the PCSA-based comparator used in this work. The PCSA comprises a differential pair (M6–M8) coupled with a latch-type comparator (M0–M5). The output of the latch is provided by inverters I1 and I2. Prior to sensing activation (SEN = ‘1’), the Dout and ($\bar{D_{\mathrm{OUT}}}$) nodes are precharged to *V*_DD_. Upon sensing activation (SAen = ‘0’), differing voltage levels on *V*_G_ and *V*_REF_ generate different discharge currents at the M6 and M7 branches. The latch generates *V*_OUT_ wherein the stronger branch turns on either M6 or M7, pulling one of the outputs ($D_{\mathrm{OUT}}$ or $\bar{D_{\mathrm{OUT}}}$) down to the ground. Figure 4b shows the timing diagram for key signals involved in the comparison between *V*_G_ and *V*_REF_.

## 4. Simulation Results

This section presents the analysis results of the STT-MRAM-based SIMPLY schemes by means of silicon-calibrated simulation models for the CMOS transistors (65 nm PDK), along with our SMTJ and DMTJ compact models reported in [26] properly modified to take into account the effects of the cryogenic operating temperature. Specifically, the compact models use temperature-dependent equations derived from experimental studies of MTJs.

The analysis is based on extensive Monte Carlo circuit-level simulations. We mainly focus on characterizing the write and read operations within the SIMPLY logic scheme.

### 4.1. Write Operation

In MTJ-based memories, write operations typically require significantly more time (and consequently consume more energy) than read accesses. While the SIMPLY scheme drastically reduces the number of write accesses, write operations are still needed for defining inputs to logical operations and potentially the output, if the MTJ state needs to be flipped. We considered the conventional STT-MTJ write scheme, utilizing two access transistors per cell for SMTJ (2T1SMTJ write configuration) and a single access transistor for DMTJ (1T1DMTJ write configuration). The two transistors in the SMTJ cell are used to avoid the source degeneration effect that can compromise successful writing to the SMTJ [26]. On the contrary, the DMTJ-based bit cell requires only one nMOS transistor per MTJ mainly due to the symmetrical switching property of the DMTJ device [26].

Table 2 shows the write pulse (*t*_write_) for a write error rate of 1 $\times \;10^{-7}$ and energy ($E_{\mathrm{write}}$) for the 2T1SMTJ and 1T1DMTJ cells operating at 300 K and 77 K. The *t*_write_ results refer to the worst-case transition between AP→P and P→AP. At 77 K, *t*_write_ increases mainly due to the rise of the critical switching current at cryogenic temperatures [29]. This effect is particularly dramatic for the SMTJ (see Table 1).

### 4.2. Reliability of Preliminary Read Operation

As detailed in Section 3, the accuracy of the SIMPLY and sFALSE operations depends heavily on the ability to reliably read the stored data. In other words, the robustness of the SIMPLY logic scheme is strongly related to the sensing margin at node *V*_G_ during the preliminary read operation. For this reason, the simulation analysis focuses on the impact of process variations on *V*_G_ during the preliminary read operation.

**Figure 5:**
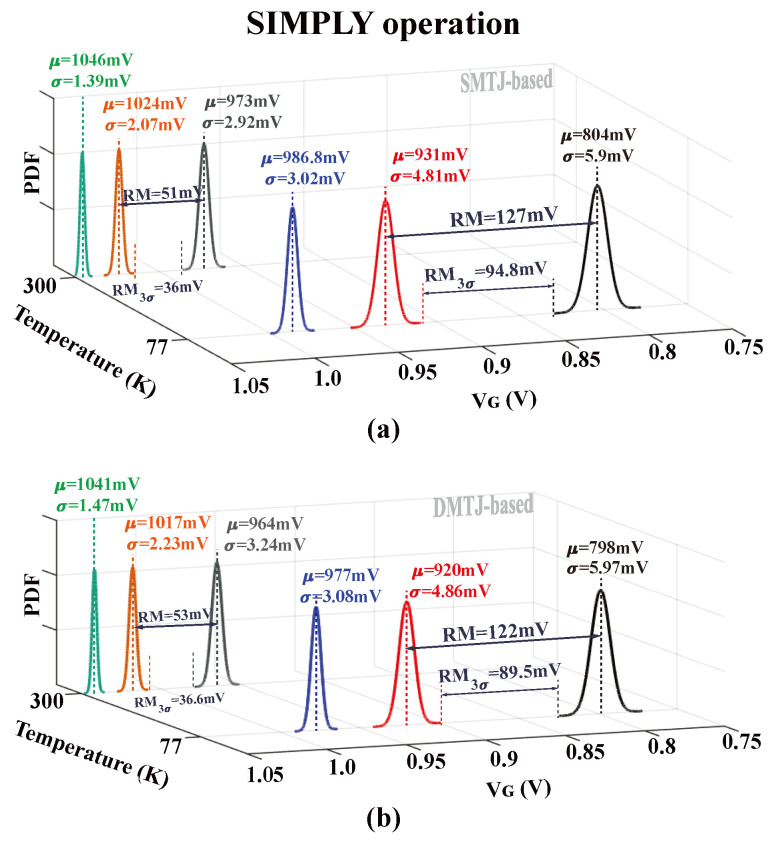
*V*_G_ statistical distributions for the conventional SIMPLY scheme based on (**a**) SMTJ and (**b**) DMTJ devices when the comparator detects **P** = **Q** = ‘0’ (gray and black lines), **P** ≠ **Q** (orange and red lines), and **P** = **Q** = ‘1’ (green and blue lines) referred to the preliminary read operation under process variations at 300 K and 77 K. Tail transistor Mn size: **Wn**/**Ln** = 1 μm/8 μm. *V*_READ_ = 1.1 V and Vbias = 1 V. *μ* represent the mean values for each case, with σ being the standard deviation of *V*_G_ distributions.

**Figure 6:**
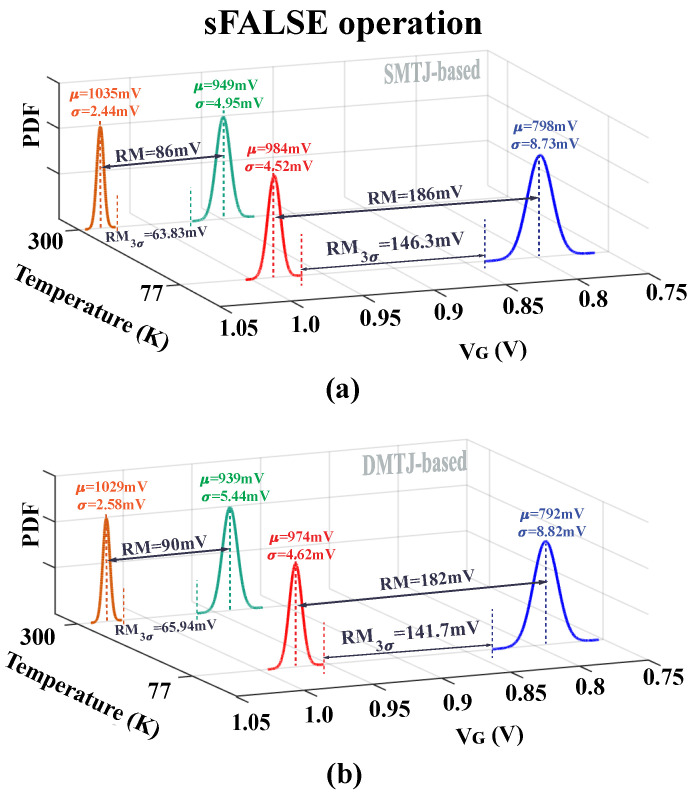
*V*_G_ statistical distributions for the sFALSE scheme based on (**a**) SMTJ and (**b**) DMTJ devices. The results are obtained when the comparator detects **Q** = ‘0’ (green and blue lines) and **Q** = ‘1’ (orange and red lines) referred to the preliminary read operation under process variations at 300 K and 77 K. Tail transistor Mn size: **Wn**/**Ln** = 1 μm/8 μm. *V*_READ_ = 1.1 V and Vbias = 1 V. μ represent the mean values for each case, with σ being the standard deviation of *V*_G_ distributions.

Figure 5 and Figure 6 show *V*_G_ statistical distributions for both DMTJ- and SMTJ-based implementations of SIMPLY and sFALSE schemes at temperatures of 300 K and 77 K. The SIMPLY results are shown for different input combinations: **P** = **Q** = ‘0’ (“gray/black” distribution in Figure 5a,b), **P**≠**Q** (“orange/red” distribution in Figure 5a,b), and **P** = **Q** = ‘1’ (“green/blue” distribution in Figure 5a,b).

The read margin (RM) is defined as the difference between the average *V*_G_ values for the **P** = **Q** = ‘0’ and **P** ≠ **Q** cases. As shown in Figure 5a, the SMTJ-based SIMPLY operating at 300 K presents an RM (RM_3σ_) of about 51 mV (36 mV). The RM improves when the SIMPLY operates at 77 K, reaching RM_3σ_ values as high as 95 mV. A similar RM_3σ_ of about 90 mV is obtained for the DMTJ-based SIMPLY operating at 77 K, as shown in Figure 5b. From Figure 6, the RM_3σ_ of the SMTJ (DMTJ)-based at 300 K and 70 K are 63.8 mV (65.9 mV) and 146.3 mV (141.7 mV), respectively. The RM_3σ_ improvement at 77 K is more than 2× compared to the 300 K operating condition.

### 4.3. Comparative Results

Table 3 summarizes the main figures of merit for the DMTJ and SMTJ SIMPLY and sFALSE schemes operating at 300 K and 77 K. The DMTJ-based (SMTJ-based) SIMPLY operation at 77 K presents RM and RM_3σ_ improvements by a factor of 2.3× and 2.4× (2.5× and 2.6×), respectively, as compared to the room temperature operation. As for the sFALSE operation, the DMTJ-based (SMTJ-based) design presents 2.2× (2.3×) higher RM_3σ_ at 77 K, as compared to the counterpart operating at 300 K. These results highlight the considerable improvement in terms of the reliability of SIMPLY LIM at cryogenic temperatures.

The higher RM comes at the cost of increased switching currents (see Table 1). This leads to a reduction in performance and an increase in consumed energy. The read energy ($E_{\mathrm{read}}$) of the DMTJ-based (SMTJ-based) scheme for the SIMPLY and sFALSE operations increases by about 1.7× and 1.4× (4.5× and 1.3×), respectively, as compared to 300 K operation. As for the write energy ($E_{\mathrm{write}}$), when operating at 77 K, the DMTJ-based scheme consumes only 4% more energy than at 300 K, while its SMTJ-based counterpart is more than twice as energy-hungry.

**Figure 7:**
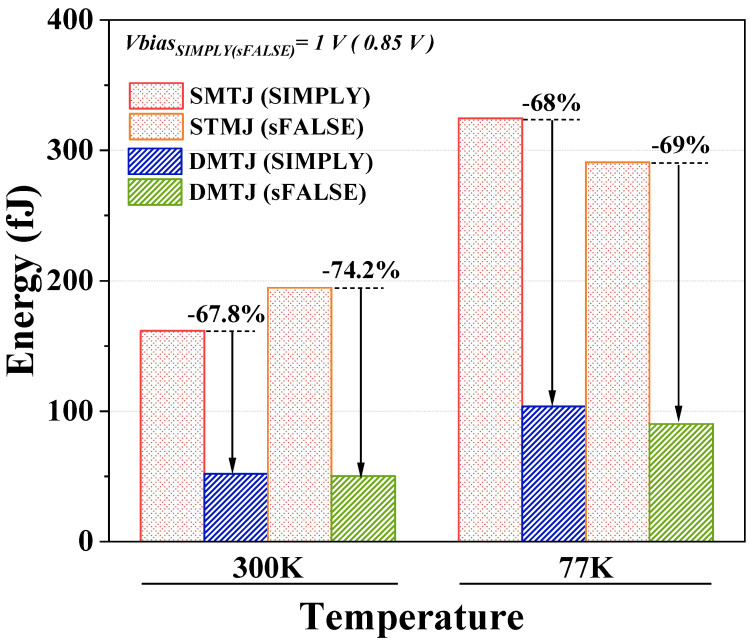
Average energy consumption for the SMTJ- and DMTJ-based logic schemes at 300 K and 77 K, for the SIMPLY and sFALSE operations.

Figure 7 compares the average energy consumption (averaged on all input combinations) of DMTJ- and SMTJ-based schemes. The DMTJ-based scheme is always the preferable option, mainly due to the lower write energy.

### 4.4. Energy Consumption of Logic Operations

We further extended our analysis to the logic-gate level, functionally evaluating SIMPLY schemes as LIM blocks performing NAND, XNOR, and XOR operations [50]. The logic operations can be implemented using the NAND logic function [51], which is generated with a sequence of SIMPLY and sFALSE operations and uses a single storage MTJ device to store the output [20].

**Figure 8:**
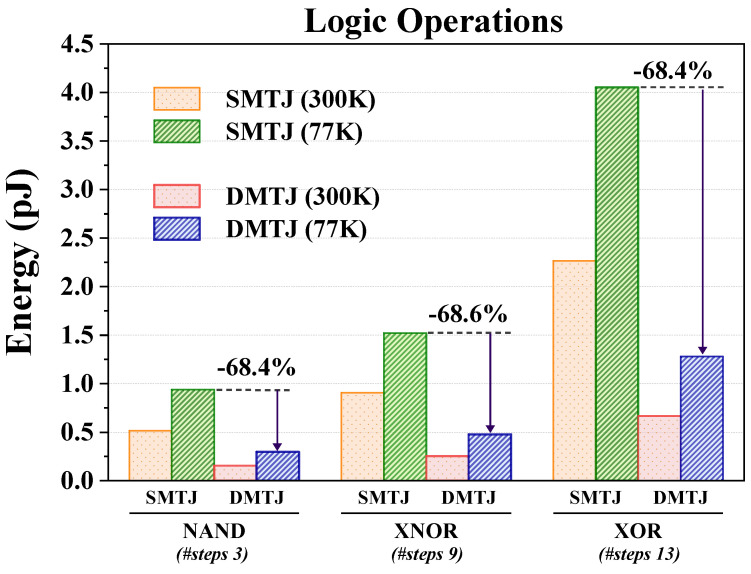
Average energy consumption for different logic operations at 77 K.

Figure 8 shows the energy consumption per logic operation under cryogenic and room temperatures for both SMTJ- and DMTJ-based schemes. The average energy consumption increases as a function of the number of required steps: 3 steps for NAND, 9 steps for XNOR, and 13 steps for XOR operation. Overall, the SMTJ-based scheme is the most energy-hungry scheme due to the higher switching currents. When compared to the SMTJ-based scheme operating at 77 K, the DMTJ-based solution consumes about 68.6% (68.4%) less energy when carrying out XNOR (XOR) operations.

### 4.5. Summary and Application Space

The Cryo-SIMPLY scheme demonstrates significant reliability enhancements at cryogenic temperatures (77 K) compared to room temperature. Conventional STT-MRAM-based SIMPLY schemes operating at room temperature suffer from high design complexity due to low sensing margins. By operating at 77 K, read sensing margins are improved, enhancing the robustness of both SMTJ- and DMTJ-based implementations, with the DMTJ-based scheme emerging as the more energy-efficient and reliable choice. The relatively high sensing margins at 77 K make reliability levels achievable at cryogenic conditions that are unattainable with conventional MTJ-based designs at room temperature. Therefore, Cryo-SIMPLY presents a compelling STT-MRAM choice for LIM applications and beyond.

Driven by the recent interest in cryogenic in-memory computing [28,42], Cryo-SIMPLY is presented as a logic-in-memory scheme tailored to low-temperature operation. Its ability to perform basic logic operations, such as NAND, XOR, and XNOR, directly within memory cells makes it ideal for data-intensive applications [52], enabling faster and more energy-efficient computation while minimizing data transfer overheads. Its efficient reconfigurable computing scheme can also be used for AI applications, in particular for binarized neural networks [53]. Lastly, Cryo-SIMPLY can be applied to energy-constrained scenarios that require highly reliable operations, such as deep space systems [27].

## 5. Conclusions

In this work, STT-MRAM-based SIMPLY schemes, operating at 77 K, were evaluated using SMTJ and DMTJ devices. The assessment relied on extensive Monte Carlo simulations, using a cryogenic-aware commercial 65 nm process and temperature-dependent Verilog-A-based MTJ compact models. Our analysis shows the DMTJ-based SIMPLY read margin at 77 K is significantly higher than at room temperature. Moreover, the evaluation at the logic-gate level highlights that, when compared to the SMTJ-based schemes, the DMTJ-based SIMPLY and sFALSE schemes provide about 69% average energy savings when performing NAND, XNOR, and XOR operations. Overall, the DMTJ-based SIMPLY scheme, operating under cryogenic conditions, is a promising solution for reliable, yet energy-efficient LIM architectures.

## Figures and Tables

**Table 1:** Parameters and characteristics of SMTJ-based and DMTJ-based devices.

Parameter	Description	DMTJ-300 K	DMTJ-77 K	SMTJ-300 K	SMTJ-77 K
*d*	Diameter	30 nm		30 nm	
** $t_{FL}$ **	FL thickness	1.2 nm		1.2 nm	
** $t_{OX,T}$ **	Top barrier thickness	0.85 nm		0.85 nm	
** $t_{OX,B}$ **	Botton barrier thickness	0.4 nm			
P	Spin polarization factor	660 m	730 m	660 m	730 m
** α **	Gilbert damping factor	30 m		30 m	
TMR(0)	Tunnel magnetoresistance	∼130%	∼200%	∼140%	∼220%
LRS	Low resistance state	15.5 kΩ	15.6 kΩ	14.2 kΩ	14.4 kΩ
HRS	High resistance state	37 kΩ	47 kΩ	35 kΩ	46 kΩ
$Ic_{P}$	Critical switching current parallel	8.6 μA	10.4 μA	30.7 μA	44 μA
$Ic_{AP}$	Critical switching current antiparallel	8.6 μA	10.4 μA	12.1 μA	13.6 μA
** Δ **	Thermal stability factor	40	175	40	216

**Table 2:** Switching characteristics for 2T1SMTJ and 1T1DMTJ cells operating at 300 K and 77 K.

	2T1SMTJ		1T1DMTJ	
**T**	**$t_{\mathrm{write}}$ (ns)**	**$E_{\mathrm{write}}$ (fJ)**	**$t_{\mathrm{write}}$ (ns)**	**$E_{\mathrm{write}}$ (fJ)**
300	8.70	301	1.56	67
77	13	458	3.28	106

**Table 3:** Comparison results.

	SIMPLY				sFALSE			
	**SMTJ**		**DMTJ**		**SMTJ**		**DMTJ**	
Temperature (K)	300	77	300	77	300	77	300	77
Vbias (V)	1	1	1	1	0.85	0.85	0.85	0.85
RM (mV)	51	127	53	122	86	186	90	182
${\mathrm{RM}}_{3\sigma }$ (mV)	36	94.8	36.6	89.5	63.8	146.3	65.9	141.7
$E_{\mathrm{read}}$ (fJ)	86.4	210.1	35.3	77.2	44.1	61.9	16.7	37.1
$E_{\mathrm{write}}$ (fJ)	301	458	67	106	301	458	67	106

## Data Availability

Data is contained within the article.

## Abbreviations

The following abbreviations are used in this manuscript:

IMC	In-memory computing
NVM	non-volatile memory
RRAM	Resistive random access memory
MRAM	Magnetoresistive random access memory
LIM	Logic-in-memory
IMPLY	Material implication
SIMPLY	Smart IMPLY
STT-MRAM	Spin-transfer torque magnetic random access memory
SMTJ	Single-barrier magnetic tunnel junction
DMTJ	Double-barrier magnetic tunnel junction
PDK	Process design kit
RL	Reference layer
${\mathrm{RL}}_{B}$	Bottom reference layer
${\mathrm{RL}}_{T}$	Top reference layer
FL	Free layer
t_OX_	Oxide barrier of thickness
LRS	Low resistance state
HRS	High resistance state
PCSA	Precharge sense amplifier
TSB	Tri-state buffers
RM	Read margin
